# Senescence-Associated Changes in Proteome and* O*-GlcNAcylation Pattern in Human Peritoneal Mesothelial Cells

**DOI:** 10.1155/2015/382652

**Published:** 2015-11-10

**Authors:** Rebecca Herzog, Silvia Tarantino, András Rudolf, Christoph Aufricht, Klaus Kratochwill, Janusz Witowski

**Affiliations:** ^1^Department of Pediatrics and Adolescent Medicine, Medical University of Vienna, 1090 Vienna, Austria; ^2^Zytoprotec GmbH, 1090 Vienna, Austria; ^3^Department of Pathophysiology, University of Medical Sciences, 60-806 Poznan, Poland

## Abstract

*Introduction*. Senescence of peritoneal mesothelial cells represents a biological program defined by arrested cell growth and altered cell secretory phenotype with potential impact in peritoneal dialysis. This study aims to characterize cellular senescence at the level of global protein expression profiles and modification of proteins with *O*-linked N-acetylglucosamine (*O*-GlcNAcylation).* Methods*. A comparative proteomics analysis between young and senescent human peritoneal mesothelial cells (HPMC) was performed using two-dimensional gel electrophoresis.* O*-GlcNAc status was assessed by Western blot under normal conditions and after modulation with 6-diazo-5-oxo-L-norleucine (DON) to decrease* O*-GlcNAcylation or* O*-(2-acetamido-2-deoxy-D-glucopyranosylidene) amino* N*-phenyl carbamate (PUGNAc) to increase* O*-GlcNAcylation.* Results*. Comparison of protein pattern of senescent and young HPMC revealed 29 differentially abundant protein spots, 11 of which were identified to be actin (cytoplasmic 1 and 2), cytokeratin-7, cofilin-2, transgelin-2, Hsp60, Hsc70, proteasome *β*-subunits (type-2 and type-3), nucleoside diphosphate kinase A, and cytosolic 5′(3′)-deoxyribonucleotidase. Although the global level of* O*-GlcNAcylation was comparable, senescent cells were not sensitive to modulation by PUGNAc.* Discussion*. This study identified changes of the proteome and altered dynamics of* O*-GlcNAc regulation in senescent mesothelial cells. Whereas changes in cytoskeleton-associated proteins likely reflect altered cell morphology, changes in chaperoning and housekeeping proteins may have functional impact on cellular stress response in peritoneal dialysis.

## 1. Introduction

Cellular senescence has emerged as a powerful biological program initiated by various forms of stress that can jeopardize the integrity of the genome. The known triggers of senescence include telomere dysfunction, oncogene activation, reactive oxygen species, and epigenomic damage [1]. By irreversibly arresting cell growth and altering cell secretory phenotype, cellular senescence plays a significant role in tumor suppression, tissue repair, and embryogenesis [2]. Senescence of peritoneal mesothelial cells has been shown to rapidly occur* in vitro*, most likely in response to culture-associated oxidative stress [3]. However, the role of mesothelial cell senescence* in vivo* is less clear.

Senescent cells show flattened and enlarged morphology and they are typically characterized by the presence of senescence-associated *β*-galactosidase (SA-*β*). Senescent cells have been detected sporadically in the peritoneal dialysis (PD) effluent and in animals infused with PD fluids [4, 5]. Moreover, senescent cells have been visualized in fresh explants of omentum from patients undergoing abdominal surgery [6].* In vitro* experiments showed that mesothelial cell senescence was accelerated by exposure to high glucose [7]. We recently found that exposure to high glucose also induced significant abundance changes of* O*-linked N-acetylglucosamine (*O*-GlcNAc) modification of mesothelial cell proteins, a posttranslational protein modification relevant in cellular survival [8]. These effects may therefore be important in the context of PD, given the extensive use of glucose as osmotic agent in PD fluids. The effect of glucose on senescence is largely related to increased oxidative stress and upregulation of transforming growth factor beta (TGF-*β*) [6, 9]. Antioxidants and anti-TGF-*β* treatments can partly reduce this effect but fail to prevent mesothelial cell senescence, still influenced by multifactorial processes not yet fully elucidated in the literature. Identification of other mechanisms could be supported by detailed characterization of the senescent mesothelial cell phenotype through technologies that determine global expression profiles of genes and proteins. Proteomics has become a standard tool in molecular biology to explore cellular mechanisms at the level of effector proteins. Two-dimensional gel electrophoresis approaches still offer the highest available resolution on the intact protein level, with the added benefit of including protein isoforms and posttranslational modifications in the global picture. The proteomic approach has already been applied to study the occurrence of senescence in other cell types [10, 11]. Therefore, in our pilot study we have attempted to analyze for the first time the changes that may occur during cellular senescence of human peritoneal mesothelial cells at the level of protein expression profiles and modification of proteins with* O*-GlcNAc.

## 2. Methods

### 2.1. Materials

Standard chemicals were purchased from Sigma-Aldrich (St. Louis, MO, USA) if not specified otherwise. NUNC (Roskilde, Denmark) tissue culture plastics were used for all cell culture procedures.

### 2.2. Mesothelial Cells

Human peritoneal mesothelial cells (HPMC) were isolated from the specimens of omentum obtained from consenting nonuremic patients undergoing elective abdominal surgery. The cells were isolated, cultured, and characterized as previously described [12, 13]. Cells were grown into senescence as detailed elsewhere [14]. Cells were considered senescent when they failed to increase in number over 4 weeks, showed enlarged morphology, and stained in majority for senescence-associated *β*-galactosidase (SA-*β*-Gal). SA-*β*-Gal was detected according to Dimri et al. [15] using a senescence *β*-galactosidase staining kit (Cell Signaling Technology (Danvers, MA, USA)).

### 2.3. Protein Sample Preparation

Young (passage 2) and senescent (passages 6–8) HPMC from 3 different donors were lysed to prepare whole cell extracts as previously described [16]; in brief cells were washed two times (250 mM sucrose, 10 mM Tris, pH 7) and lysed by incubation with 800 *µ*L lysis buffer (30 mM Tris, pH 8.5, 7 M urea, 2 M thiourea, 4% 3-[(3-cholamidopropyl) dimethylammonio]-1-propanesulfonate (CHAPS), 1 mM ethylenediaminetetraacetic acid (EDTA), one tablet of complete protease inhibitor (Roche, Basel, Switzerland), and one tablet of PhosStop phosphatase inhibitor (Roche) per 100 mL) per 75 cm^2^ culture flask for 10 min at 25°C. The resulting lysates were stored at −80°C until further processing. Total protein concentration was determined by the 2D Quant kit (GE Healthcare, Uppsala, Sweden) according to the manufacturer's manual.

### 2.4. Two-Dimensional Gel Electrophoresis

50 *μ*g of total protein per sample, in triplicates, was brought to a final volume of 210 *μ*L with rehydration buffer (5 M urea, 0.5% CHAPS, 0.5% Pharmalyte (Bio-Rad, Hercules, CA, USA), and 12 *μ*L/mL of DeStreak reagent (GE Healthcare)) and subsequently applied on immobilized pH gradient (IPG) strips (ReadyStrip pH 3–10, nonlinear, 11 cm, Bio-Rad). The strip was covered with silicone oil, actively rehydrated (50 V, 12 h, 20°C), and then focused (Bio-Rad Protean I12) by increasing the voltage to 5000 V (total 50 kVh, current limit 30 *μ*A/strip). Focused strips were stored at −80°C until further use. Before second dimension, each strip was incubated twice for 20 min in 2 mL equilibration buffer (6 M urea, 2% (w/v) sodium dodecyl sulfate (SDS), 25% glycerol, and 3.3% 50 mM Tris/HCl pH 8.8, stained with bromophenol blue) first supplemented with 10 mg/mL dithiothreitol (DTT) and then 48 mg/mL 2-iodoacetamide (IAA). The second dimension was carried out using precast Criterion TGX Stain-Free polyacrylamide gels (133 × 87 × 1 mm, Bio-Rad) on a Criterion cell (Bio-Rad) for 2 hours at 20 mA.

### 2.5. Visualization and Analysis of Proteins

Protein spots were visualized utilizing the ChemiDoc XRS system (Bio-Rad) by UV-induced reaction. Gel images were acquired and processed using Image Lab software (Bio-Rad). The images were analyzed using the Delta2D 4.5 software (Decodon GmbH, Greifswald, Germany) with group-wise image alignment and spot detection on the resulting fused image. Protein identifications of the mesothelial cell proteome from our recent work [17] accomplished by mass spectrometry were reassigned from the original images to the master image of the current study.

### 2.6. *In Vitro* Treatment with Modulators of* O*-GlcNAcylation

HPMC were seeded onto 12-well culture plates and incubated for 48 hours with chemical inhibitors of the hexosamine biosynthesis pathway (HBP) 6-diazo-5-oxo-L-norleucine (DON) to decrease* O*-GlcNAc abundance or O-(2-Acetamido-2-deoxy-D-glucopyranosylidene) amino* N*-phenyl carbamate (PUGNAc) to inhibit removal of* O*-GlcNAc and therefore increase* O*-GlcNAc abundance. After the treatment, cells were washed and lysed as described above.

### 2.7. Western Blot and ELISA

Cell extracts were prepared as described above, and equal amounts of total protein were separated by SDS-PAGE on a Bio-Rad Criterion cell using Criterion precast gels of 1 mm thickness (Bio-Rad). Proteins were electroblotted onto polyvinylidene fluoride (PVDF) membranes (Millipore, Billerica, MA, USA) immediately after the run by tank blotting using a Criterion blotting cell (Bio-Rad) and the respective transfer buffer (200 mM glycine, 25 mM Tris, 0.1% SDS, and 20% methanol). Membranes were blocked with 5% bovine serum albumin (BSA) and incubated with an antibody against* O*-GlcNAc (RL2, Abcam, Cambridge, UK) over night at 4°C. After incubation with the secondary, peroxidase-coupled antibody (Polyclonal Rabbit Anti-Mouse Ig/HRP P0260; DakoCytomation, Carpinteria, CA) detection was accomplished by using enhanced chemiluminescence solution (Western Lightning reagent; Perkin Elmer, Boston, MA) and a ChemiDoc XRS chemiluminescence detection system (Bio-Rad). Densitometric quantification was accomplished using the Image Lab software (Bio-Rad).

### 2.8. Statistics

Statistical analyses were performed using SPSS 17 (SPSS Inc., Chicago, IL, USA) and Sigmaplot 11.0 (Systat Software GmbH, Erkrath, Germany). Values from different groups were compared using *t*-tests or ANOVA where appropriate. In case of ANOVA Tukey's HSD was used as post hoc test. *p* values lower than 0.05 were considered significant. The results are presented as means ± SEM.

## 3. Results

**Figure 1:**
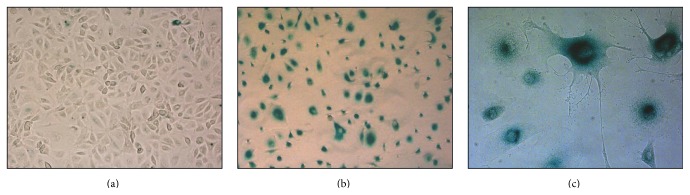
Morphology and senescent phenotype of HPMC. Expression of senescence-associated *β*-galactosidase (SA-*β*-Gal) was compared by light microscopy and SA-*β*-Gal staining in young (a) and senescent ((b) and (c)) HPMC. Magnification (a)-(b): 10x and (c) 40x.

As previously described [14], serial passages of HPMC led to a gradual decline in cell proliferative capacity and to the development of senescent phenotype characterized by altered morphology and extensive staining for SA-*β* (Figure 1).

### 3.1. Proteomics Analysis of Total Protein Extracts of Young and Senescent HPMC

**Figure 2:**
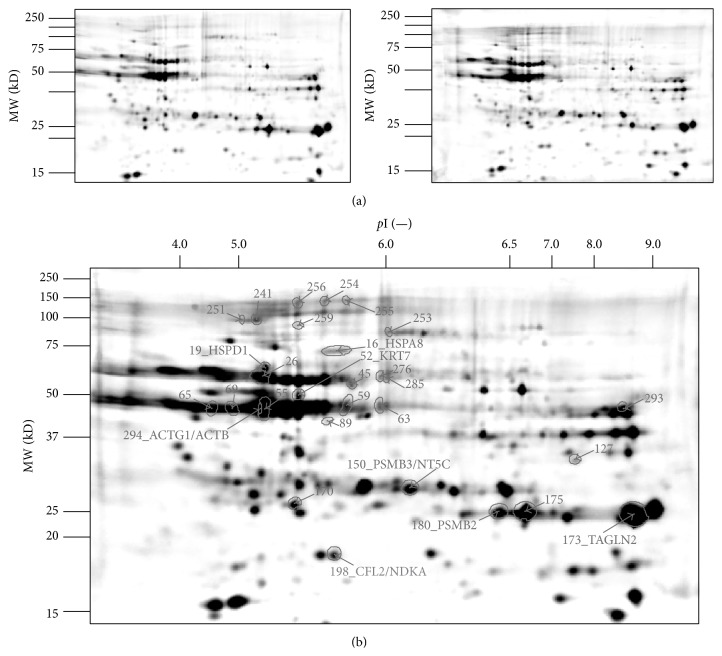
2D Gel images of young and senescent HPMC. (a) Representative 2D Gel of senescent (left panel) and young (right panel) human peritoneal mesothelial cells (HPMC). (b) Fusion image of 2D protein pattern of senescent and young HPMC (total spot count: 305). Protein spots found statistically significant altered (*p* < 0.05) in the comparison between senescent and young (*n* = 29) are marked with spot boundaries, spot label, and name of identified proteins (*n* = 11).

To assess the effects of cellular senescence with the aid of comparative proteomics techniques, three technical replicate gels per group (representative gel images in Figure 2(a)) were analyzed and compared using Delta2D 4.5 software (Decodon GmbH). Group-wise image alignment and spot detection on the resulting fused image revealed a common spot pattern of 305 protein spots (Figure 2(b)).

**Figure 3:**
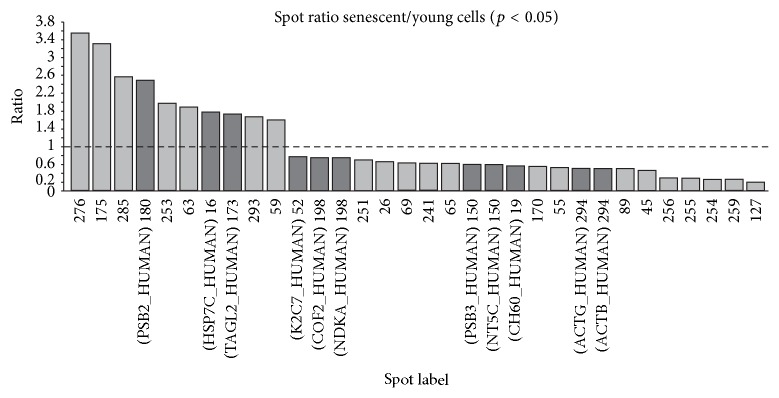
Spot abundance ratio of significantly altered spots (*p* < 0.05) between young and senescent HPMC. Changes in spot abundance are represented as spot volume ratio for the 29 significantly altered spots found in the comparison between young and senescent human peritoneal mesothelial cells (HPMC).

Quantitative spot analysis of the young and senescent cell proteome revealed 29 spots differing significantly in abundance (*p* ≤ 0.05) (Figure 2(b)).  Figure 3 shows the senescent/young spot ratio for each spot identified. Of those, the abundance of 10 (34%) and 19 (66%) proteins in senescent cells was found to be increased and decreased, respectively.

Based on protein identifications made in previous studies [17, 18] we were able to identify 11 unique proteins shown in Figure 2(b): actin (ACTG and ACTB), cytokeratin-7 (KRT7), cofilin-2 (CFL2), transgelin-2 (TAGLN2), Hsp60 (HSPD1), Hsc70 (HSPA8), proteasome subunits beta (PSMB2 and PSMB3), NDK A (NME1), and dNT-1 (NT5C).

Interestingly, the majority of these proteins are known to be involved in cellular processes that can be modulated by* O*-GlcNAcylation. Characteristics of individual proteins, their potential (number of serine and threonine residues), and predicted (based on the bioinformatic algorithm* O*-GlcNAc-Scan [19])* O*-GlcNAc modification sites as well as references to experimentally validated* O*-GlcNAc modification sites of these proteins are listed in Table 1.

### 3.2. *O-*GlcNAc Dynamics under Chemical Modulators

**Figure 4:**
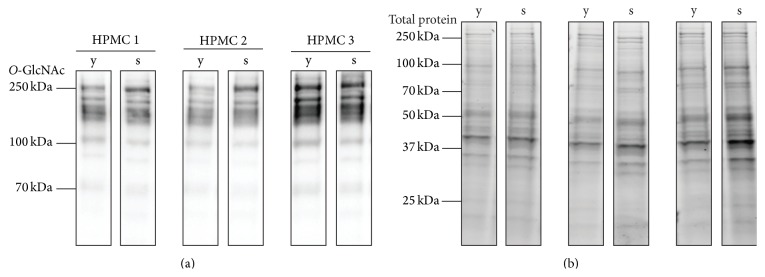
*O*-GlcNAc western blot of young and senescent HPMC. (a)* O*-GlcNAc specific western blot of young (y) and senescent (s) human peritoneal mesothelial cells (HPMC) from three different donors (HPMC 1–3). (b) Corresponding total protein loading (stain-free technology Bio-Rad).

**Figure 5:**
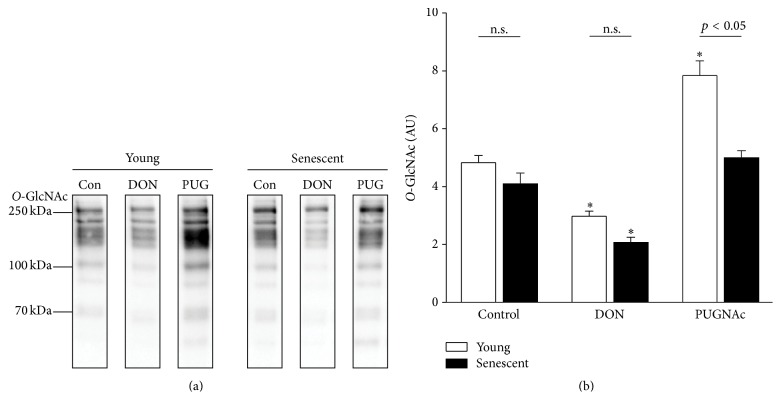
*O*-GlcNAc western blot of young and senescent HPMC treated with modulators of the hexosamine biosynthesis pathway (HBP). (a) Representative* O*-GlcNAc specific western blot of young (left) and senescent (right) human peritoneal mesothelial cells (HPMC) treated with* O*-GlcNAcylation inhibitor (DON) or an inhibitor of the* O*-GlcNAcase (PUG = PUGNAc). (b) Densitometric analysis of western blots for effects of DON and PUGNAc on* O*-GlcNAcylation (*n* = 3). n.s., not significant; ^*∗*^ *p* < 0.05 versus control.

Direct comparison of cellular proteins from young and senescent mesothelial cells shows similar levels of global* O*-GlcNAcylation (Figure 4). Nevertheless, modulation of* O*-GlcNAcylation by chemical inhibitors of the HBP revealed marked differences between the two cell statuses. The addition of DON, a glutamine fructose-6-phosphate amidotransferase (GFAT) inhibitor, resulted in a decrease of* O*-GlcNAc levels in both young and senescent cells (61.6%  ± 3.7 versus 42.9%  ± 3.7, resp.) (Figure 5). In contrast, addition of the* O*-GlcNAcase inhibitor PUGNAc resulted in increased* O*-GlcNAc levels in young but not in senescent cells (162.2%  ± 10.6 versus 103.5%  ± 5.0, resp.). Thus the* O*-GlcNAc status under control conditions showed a higher relative level in senescent than in young mesothelial cells.

## 4. Discussion

In this study we show that the senescent phenotype of mesothelial cells is associated with quantitative changes in the cellular proteome. These changes are seen predominantly in cytoskeleton-associated proteins but also in chaperoning and housekeeping proteins. Whereas changes in cytoskeletal proteins are likely to contribute to the altered senescent cell morphology, changes in the chaperone protein family may have functional impact on cellular stress responses. In this respect, previous studies have clearly demonstrated the importance of stress responses in mesothelial cells exposed to PD fluids [17, 18]. The global level of* O*-GlcNAcylation showed comparable levels in young and senescent mesothelial cells; however, senescent cells were not able to increase their level of global protein* O*-GlcNAcylation, suggesting altered dynamics of* O*-GlcNAc regulation.

While many studies have clearly documented phenotypic changes in cell senescence, they have also revealed that the course and the rate of senescence very much depend on the cell type and on the specific pathophysiological setting. In this respect, senescence of HPMC in culture displays some interesting features [3], including a rather swift and sudden loss of proliferative capacity, extensive DNA damage in non-telomeric regions, and high susceptibility to oxidative stress. In the present study we have been able to further characterize this phenotype by identifying a set of proteins altered in their expression in senescent cells.

About half of the senescence-induced changes were identified in proteins involved in cytoskeletal organization. These included two isotypes of actin and cytokeratin, key structural proteins with major biological roles in early and late cellular development phases and status [20–22]. The actin-binding proteins cofilin and transgelin are described to be crucial regulators of actin dynamics [23]. Cofilin promotes actin filament elongation as an actin-severing protein [24]. Transgelin is a shape-change sensitive actin cross-linking/gelling protein found to be overexpressed as well in senescent fibroblasts [25–27]. The next functional group identified is composed of proteins that are key players in cellular stress response. They are exemplified by Hsp60 and Hsc70, which are molecular chaperons known to be involved in cellular repair, transport, and protein metabolism [28]. These processes are essential for cellular survival under both control and stress conditions and their role in senescence has recently been reviewed [29–31]. Similarly, changes in the abundance of the proteasome-associated proteins may be linked to altered proteolytic activities and proteasome content that have been reported to occur in senescent cells [32–34]. It has been shown that proteasome inhibition results in the appearance of a senescence-like phenotype in fibroblasts [35]. However, the number of proteins identified as differently expressed by senescent mesothelial cells may be viewed as relatively modest, which may point at other regulatory mechanisms modulating the protein activity in senescence. They may include posttranslational protein modifications, protein-protein interactions and networking, protein trafficking, and cellular localization.

In this respect it is interesting to note that all but one of these proteins have been previously reported to be potentially* O*-GlcNAcylated proteins and/or belong to protein families with* O*-GlcNAc modified members [24, 36–45]. This suggests that senescence-associated processes may be regulated partly by* O*-GlcNAc modifications.* O*-GlcNAcylation is a ubiquitous posttranslational mechanism regulated by nutrient availability and enzyme activity [46]. It has been estimated that at least 3,000 proteins can be modified by* O*-GlcNAc [47].

Although the assessment of global* O*-GlcNAcylation did not demonstrate significant differences between young and senescent cells under control conditions, pharmacological intervention led to significant changes in the dynamics of early versus late-passage mesothelial cells. These data supplement current knowledge of* O*-GlcNAc cycling during cell senescence and suggest that changes in* O*-GlcNAc dynamics in senescence may be more important than the global level of* O*-GlcNAcylation.* O*-GlcNAc modification of individual target proteins may thus contribute to senescence by modulating cytoskeletal organization, stress response, and proteasome activity.

In this respect,* O*-GlcNAcylation was reported to impact on structural and regulatory proteins of the cytoskeleton [46], modulating their solubility and preventing the aggregation of denatured proteins [48]. Dynamics in* O*-GlcNAc have been described in key players of the cellular architecture such as actin, cytokeratins, and actin-binding proteins [37]. For example, specific changes in the* O*-GlcNAcylation of cytokeratins have been reported to occur during cell cycle progression [37]. They probably modulate solubility of cytokeratins [36]. Recent studies also suggested a functional role for* O*-GlcNAcylation of cofilin in regulating actin dynamics, as silencing of cofilin by siRNA abolished* O*-GlcNAc transferase- (OGT-) enhanced cell mobility [24].

*O*-GlcNAcylation was also found to be involved in cellular stress response pathways [48]. For example,* O*-GlcNAcylation can regulate the key heat shock transcription factor HSF-1 and thus impact on subsequent expression of several heat shock protein (Hsp) families [49]. Enhanced* O*-GlcNAcylation of cytosolic Hsp60 was shown to be associated with decreased interactions between Hsp60 and Bax, resulting in translocation of Bax to mitochondria and leading to cell death [42]. This aspect might be relevant in the context of peritoneal dialysis (PD), as* O*-GlcNAcylation of Hsp60 was found to be upregulated by high glucose [39, 42]. On the other hand, increased* O*-GlcNAcylation of other proteins may promote cellular protection by increasing their binding to Hsp70 [48].

*O*-GlcNAcylation may also regulate proteasome function under control and stressful conditions [46]. Recent publications suggest that proteasome activity and cellular energy status might be coupled to* O*-GlcNAcylation, acting as a metabolic sensor: an increase in nutrient-dependent posttranslational modification of the proteasome was shown to correlate with decreased proteasome activity and protein degradation [50].

Taken together, our results add to recent literature describing association of* O*-GlcNAcylation with changes in proteins in aging [51–53]. In this respect, increased* O*-GlcNAc levels were found in vital tissues of aged rodents [51]. In* Caenorhabditis elegans*, genetic manipulation of enzymes regulating* O*-GlcNAcylation resulted in changes in life span and resistance to stress [53]. In that system, several hundred promoters, including those involved in aging, were found to display differential cycling of* O*-GlcNAc [52]. Thus, linking* O*-GlcNAc cycling to higher order protein structures may provide insights into how cells respond to potential stressors and inducers of senescence. In mesothelial cells, senescence will likely influence stress response to glucose-based PD fluids [8].

In addition to antibody-based detection techniques of global* O*-GlcNAc-pattern identification, recently more and more studies emerged, investigating into sequence-specific localization of functional alterations caused by protein modification with* O*-GlcNAc. While these methods certainly will provide an important leap in understanding complex biological regulatory circuits, the analytical techniques, mainly relying on mass spectrometry combined with soft ionization methods, are still more suitable for focusing on an individual candidate protein than on global effects [44, 54]. Nevertheless, future studies will have to integrate these techniques for more detailed description of senescence-associated changes in specific* O*-GlcNAcylation.

We have previously demonstrated that, in primary peritoneal mesothelial cells, cultured from human omentum or from clinical effluent of PD patients, basal* O*-GlcNAc levels were in an intermediate range and sensitive to modulation (as confirmed in the present study for young cells). Exposure to commercially available PD fluids increased the global* O*-GlcNAc status close to maximum levels induced by PUGNAc. By testing single PD fluids components we showed that the increase of* O*-GlcNAcylation was mainly driven by glucose [8]. Chemical modulation of* O*-GlcNAc levels led to corresponding changes in HSP expression and cellular viability. These experiments suggested that the cytoprotective effect of the dipeptide alanyl-glutamine toward peritoneal mesothelial cells [8, 16] could be related to its ability to modulate* O*-GlcNAcylation. However, further studies are required to investigate in detail the consequences of senescence-associated changes in* O*-GlcNAcylation for mesothelial cell response to PD fluid. These studies will also need to assess the complex interplay of* O*-GlcNAc with specific target proteins involved in cellular senescence and to further define the specific pattern of individual mesothelial proteins that are* O*-GlcNAcylated in young and senescent cells in response to PD fluid exposure.

## Figures and Tables

**Table 1:** Identified proteins showing significant differential abundance between young and senescent HPMC cells (*p* < 0.05) with references of their predicted or reported *O*-GlcNAcylated sites.

Protein name	Gene name	SwissProt entry name	MW (kD)^a^	*p*I^b^	Length^c^	Spot^d^	Potential *O*-GlcNAc sites^e^	S^e^	T^e^	Predicted *O*-GlcNAc sites^f^	S^f^	T^f^	References^g^
Actin, cytoplasmic 2	ACTG1	ACTG_HUMAN	41.8	5.31	375	294	51	25	26	9	8	1	[43]
Actin, cytoplasmic 1	ACTB	ACTB_HUMAN	41.7	5.29	375	294	51	25	26	9	8	1	[38, 40, 44]
Keratin, type II cytoskeletal 7	KRT7	K2C7_HUMAN	51.4	5.39	469	52	63	46	17	4	4	0	—^*∗*^ [36–38, 44]
Cofilin-2	CFL2	COF2_HUMAN	18.7	7.88	166	198	20	12	8	4	4	0	[24]
Transgelin-2	TAGLN2	TAGL2_HUMAN	22.4	8.45	199	173	17	7	10	2	2	0	[41, 43]
60 kDa heat shock protein, mitochondrial	HSPD1	CH60_HUMAN	61.0	5.24	573	19	14	6	8	6	4	2	[37, 39, 41, 42, 44]
Heat shock cognate 71 kDa protein	HSPA8	HSP7C_HUMAN	70.9	5.37	646	16	82	35	47	4	2	2	[36–38, 41, 43–45]
Proteasome subunit beta type-2	PSMB2	PSB2_HUMAN	22.8	6.52	201	180	18	10	8	9	5	4	[41, 43]
Proteasome subunit beta type-3	PSMB3	PSB3_HUMAN	22.9	6.12	205	150	20	7	13	2	0	2	—^*∗*^ [41, 43]
Nucleoside diphosphate kinase A	NME1	NDKA_HUMAN	17.1	5.82	152	198	11	6	5	3	2	1	[41]
5′(3′)-deoxyribonucleotidase, cytosolic type	NT5C	NT5C_HUMAN	23.4	6.18	201	150	14	6	8	1	0	1	—

^a^Relative molecular mass of the protein as calculated from the amino acid sequence of the polypeptide without any co- or posttranslational modifications; ^b^calculated *p*I of the protein as obtained from SwissProt database; ^c^number of amino acids in the protein sequence; ^d^spots numbered according to Figure 2; ^e^potential *O*-GlcNAcylation sites expressed as the total number of serine (S) and threonine (T) residues in the protein sequence; ^f^predicted *O*-GlcNAcylation sites expressed as the number of predicted serine (S) and threonine (T) residues in the protein, obtained from dbOGAP database using default settings in OGlcNAcScan tool; ^g^references within protein candidates that have been reported to be *O*-GlcNAcylated. ^*∗*^proteins from the same protein family of the candidate have been reported to be *O*-GlcNAcylated. [dbOGAP: http://cbsb.lombardi.georgetown.edu/hulab/OGAP.html].
